# Substance P Increases Cell-Surface Expression of CD74 (Receptor for Macrophage Migration Inhibitory Factor): In Vivo Biotinylation of Urothelial Cell-Surface Proteins 

**DOI:** 10.1155/2009/535348

**Published:** 2009-03-22

**Authors:** Katherine L. Meyer-Siegler, Shen-Ling Xia, Pedro L. Vera

**Affiliations:** ^1^Research & Development Service, Bay Pines VA Healthcare System, VA Medical Center, Bay Pines, FL 33744, USA; ^2^Department of Molecular Medicine, University of South Florida, Tampa, FL 33620, USA; ^3^Research Service, North Florida/South Georgia Veterans Health System; Department of Medicine, University of Florida College of Medicine, Gainesville, FL 32608, USA; ^4^Division of Urology, Department of Surgery, University of South Florida, Tampa, FL 33620, USA

## Abstract

Macrophage migration inhibitory factor (MIF), an inflammatory cytokine, and its receptor CD74 are upregulated by bladder inflammation. MIF-mediated signal transduction involves binding to cell-surface CD74, this study documents, in vivo, MIF-*CD74* interactions at the urothelial cell surface. N-hydroxysulfosuccinimide biotin ester-labeled surface urothelial proteins in rats treated either with saline or substance P (SP, 40 *μ*g/kg). The bladder was examined by histology and confocal microscopy. Biotinylated proteins were purified by avidin agarose, immunoprecipitated with anti-MIF or anti-CD74 antibodies, and detected with strepavidin-HRP. Only superficial urothelial cells were biotinylated. These cells contained a biotinylated MIF/CD74 cell-surface complex that was increased in SP-treated animals. SP treatment increased MIF and CD74 mRNA in urothelial cells. Our data indicate that intraluminal MIF, released from urothelial cells as a consequence of SP treatment, interacts with urothelial cell-surface CD74. These results document that our previously described MIF-CD74 interaction occurs at the urothelial cell surface.

## 1. Introduction

The urothelium is no longer regarded as a passive barrier but rather as a complex “sensory web” transducing signals from inside the viscus (and in response to neuronal and local changes) through the activation of cell-surface receptors [1]. Therefore, the study of interactions between urothelial cell-surface receptors expressed during normal and diseased states and different molecules is likely to shed light on urothelial signal transduction in health and disease.

A central question in the study of tissue response to inflammation is how cells titrate and integrate cytokine signals in order to respond in an appropriate manner. Regulation of the specific pathway through bioavailability of ligands and/or receptors is one potential mechanism. Receptor bioavailability is achieved predominantly through the cell-surface expression of specific functional receptors. The ligand-receptor interaction results in the transduction of these extracellular signals across the plasma membrane and, through the activation of intracellular signaling pathways, affect the appropriate functional response.

We have been studying the role of a unique proinflammatory cytokine, macrophage migration inhibitory factor (MIF) in experimental and clinical cystitis [2–4]. MIF is constitutively expressed and secreted by numerous cell types, and significant levels can be found in blood, urine, cerebrospinal and other body fluids [5, 6]. Therefore, circulating or extracellular MIF is readily available, even under basal (“normal”) conditions [7]. The ubiquitous nature of this cytokine suggests that cellular response to MIF is dictated either by increasing the amount of available MIF or by changes in receptor bioavailability. We have demonstrated that subcutaneous SP increases intraluminal MIF amounts, which is localized in preformed urothelial stores [4]. In addition, sequestering intraluminal MIF with antibodies reduced SP-induced inflammatory changes in the rat bladder [8]. Thus, increased intraluminal MIF participates in a feedback loop that continues to maintain SP-induced inflammatory changes in the bladder. The precise mechanism of MIF action is unknown. However, numerous studies have contributed to the current consensus that the involvement of MIF is central in the initiation of the inflammatory response [9, 10].

Using in vitro models, the MIF receptor was identified as the cell-surface form of CD74 (also known as the invariant chain) [11]. The primary function of CD74 is to regulate peptide loading onto major histocompatibility class II heterodimers [12]. However, a small portion of the total cell CD74 content is expressed on cell surfaces, but the exact function of cell-surface CD74 is not known [13]. Recent studies have documented the interaction of MIF with CD74; however, it is not likely that this interaction is sufficient to produce proinflammatory effects without the presence of CD44 (MIF-CD74 signaling component) [11, 14]. MIF, CD74, and CD44 are all upregulated during experimental bladder inflammation suggesting that MIF-mediated signaling may be involved in cystitis [15, 16]. Generally, cell-surface expression of CD74 is rather low, varying in monocytes from a few hundred to a few thousand molecules per cell (for comparison, there are approx. 3 × 10^5^ molecules of the MIF signal molecule CD44 per cell) again suggesting that cell-surface availability of CD74 is the rate limiting variable in MIF signal transduction [17].

We previously showed that additional MIF is released from the bladder as part of the inflammatory response, and is subsequently localized to umbrella cells in the urothelium [18]. In addition, we showed increased amounts of CD74 protein localized to the urothelial layer suggesting that CD74 is a potential receptor for MIF-mediated signal transduction during bladder inflammation [16]. Therefore, we hypothesize that urothelial cell-surface CD74-MIF binding and subsequent association with CD44 activates ERK1/2 signal transduction pathways. Taken together, our previous results suggest that the bioavailability of the CD74 rather than MIF dictates cell response. Therefore, we examined the cell-surface location and extents of CD74 and MIF-CD74 in urothelial cells and the effects of bladder inflammation induced by SP using in vivo biotinylation of cell-surface proteins and confocal microscopy.

## 2. Methods

### 2.1. Animal Model

In vivo biotinylation of urothelial cell-surface proteins—twelve male Sprague-Dawley rats (250–300 gm) were anesthetized with sodium pentobarbital (60 mg/kg; i.p.) and divided into two groups: (1) saline (*N* = 6): bladders isolated from kidneys by cutting ureters; bladders emptied of urine, rinsed twice with PBS, and replaced with 0.3 mL biotinylation reagent (1 mg/mL N-hydroxysulfosuccinimide biotin ester, Pierce Biochemicals, Rockford, Ill, USA); saline treatment (s.c.); (2) substance P (SP, Sigma, St. Louis, Mo, USA; *N* = 6): bladders treated as in group (1) with SP treatment (40 *μ*g/kg; s.c.). After 1 hour, the bladders were excised and cut in half. Half of the bladder was placed in formalin for histology and confocal microscopy, while the remaining piece was carefully stretched and urothelial cells enriched using epithelial aggregate separation and isolation (EASI) [19].

### 2.2. Histology

Biotinylation of bladder tissue was detected by exposing frozen bladder sections (14 *μ*m thick; intact bladders) to strepavidin- horseradish peroxidase (HRP) conjugate and developing with 1% 3, 3′-diaminobenzidine tetrahydrochloride in 0.3% hydrogen peroxide (Sigma). Frozen sections (14 *μ*m thick) of scraped bladders were stained using hematoxylin to document that only the epithelium was removed from the underlying tissue.

### 2.3. Purification and Detection of Biotinylated Proteins

Isolated urothelial cells were collected by centrifugation at 10 000 g for 5 minutes, then washed in three times with 100 mM glycine in PBS pH 8.0 to inactivate residual biotinylation reagent. Half of the collected urothelial cells were processed for protein and the remaining used for RNA isolation.

Proteins were extracted from isolated urothelial cells by resuspension of washed cells in ice-cold RIPA buffer (50 mM Tris-HCL, pH 7.5, 150 mM NaCl with 1% Triton X-100, 0.1% SDS, and 0.25% sodium deoxycholate, plus general protease inhibitors; Sigma), dounce homogenization followed by centrifugation at 12 000 g for 15 minutes at 4°C. Protein concentrations in the cleared cell lysates were determined using the Micro-BCA protocol (Pierce Biochemicals, Rockford, Ill, USA).

Urothelial cell lysates (10 *μ*g total protein) were separated on 4–12% bis tris acrylamide gels (NuPAGE, Invitrogen, Carlsbad, Calif, USA) and transferred to PVDF. Western-blotting using antivimentin (fibroblast marker, Neuromics, Edina, Minn, USA; *#*CH23010) verified that only urothelial cells were removed and isolated. Western blots were stripped (0.2 M glycine, pH 2.2 containing 0.1% SDS, 1% Tween-20) and reprobed with GAPDH antibody (Santa Cruz Biotechnology, Santa Cruz, Calif, USA; sc-20357), which served as a loading control.

Urothelial surface proteins labeled with biotin were purified from total urothelial cell proteins by avidin-agarose affinity chromatography (A1979, Sigma). Nonexchangeable biotin binding sites were blocked by incubating 100 *μ*L of avidin-agarose resin with 400 *μ*L of biotin (1 mg/mL) for 15 minutes at room temperature. Biotin was eluted from exchangeable binding sites by washing the resin three times with 500 *μ*L 0.1 M glycine, pH 2.0. The prepared resin was equilibrated with PBS and incubated with 1 mg of total urothelial cell lysate. Biotinylated proteins were eluted with 2 mM biotin and concentrated fivefold through 0.2 *μ*m filters. Avidin-purified proteins (15 *μ*L of fivefold-concentrated avidin-agarose eluate) isolated from each animal were separated by SDS-PAGE (Invitrogen) and transferred to PVDF. Blots were blocked in blocking buffer (1% BSA, 10 mM PBS, pH 7.5. and 0.05% Tween-20) for 1 hour at 37°C and total biotinylated protein detected by incubating for 1 hour at 37°C with HRP-labeled strepavidin (1:200 dilution in blocking buffer R&D Systems, Minneapolis, Minn, USA). The blot was then washed, and biotinylated proteins visualized and quantified with a chemiluminescent imaging system (substrate, Super Signal West Dura, Pierce; Kodak Image Station, Kodak, Rochester, NY, USA). Total band intensities were determined using the manufacturer software (Kodak) and expressed in arbitrary units. Data are expressed as median ± interquartile range.

### 2.4. Immunoprecipitation

Neither MIF nor CD74 could be detected in the avidin-purified urothelial cell lysates using standard Western blotting procedures, therefore immunoprecipitation was used. Equal amounts of total protein form the urothelial cell lysates (1 mg, saline animals 4 and 5; SP animals 9 and 10) were purified by avidin agarose, and the resulting biotinylated proteins precleared by incubation with protein A agarose beads for 1 hour at 4°C. The precleared biotinylated proteins were divided into two equal aliquots and incubated overnight with 5 *μ*g of the corresponding antibody (CD74, Santa Cruz Biotechnology, sc-5438; or MIF, R&D Systems, AF-289-PB) at 4°C. Protein A-agarose beads (50% slurry) were added to the mixture, which was incubated for 1 hour at 4°C. Beads were then washed three times in PBS and then resuspended in SDS sample buffer. Controls included incubation of precleared samples with nonspecific goat IgG (Sigma), GAPDH antibody (Santa Cruz Biotechnology, sc-20357), or samples without the addition of antibody in the immunoprecipitation reaction. The resulting immunoprecipitated protein was separated by 4–12% Bis-Tris SDS-PAGE (Invitrogen) and transferred to PVDF. Incubating the blot overnight with streptavidin-HRP conjugate (R&D Systems, 1:200 dilution in blocking buffer, no detection or secondary antibody was used) was used to visualize only immunoprecipitated biotinylated protein. Bands were visualized as described above.

### 2.5. Coimmunoprecipitation

Cell-surface MIF-CD74 complexes were isolated by incubating avidin-agarose purified biotinylated proteins (purified from 1 mg total urothelial lysate) with MIF antibody (R&D Systems, 1:1000 dilution, saline animals 1, 2, 3, and 6; SP animals 7, 8, 11, and 12) at 4°C overnight. Immunoprecipitated proteins were separated as described above, and CD74 containing MIF complexes were detected by overnight incubation with anti-CD74 antibody (Santa Cruz Biotechnology, sc-5438) followed by an antigoat HRP conjugate (Pierce) and visualized as described above.

### 2.6. PCR

Urothelial cell total RNA was isolated using TriZol reagent (Invitrogen) with 1 *μ*g of RNA reverse transcribed and the resulting cDNA (2 *μ*L) used for relative quantitative PCR. Relative quantitative PCR conditions for *MIF* and *CD74* were established by determining the linear range of the target. Conditions for the 18S rRNA primer/competimer ratio were determined as described by the manufacturer (3:7 primer:competimer ratio, Ambion, Austin, Tex, USA). These primer rations optimized PCR conditions allow the generated 18S rRNA product to be used as an internal standard to correct for variation in the initial amount of total RNA reverse transcribed, as well as tube-to-tube variations inherent in the PCR reaction. *MIF* and *CD74* were amplified along with the 18S rRNA internal standard using high stringency conditions: 30 cycles of 94°C 1 minute, 63°C 1 minute, 72°C 1 minute as described previously [20]. PCR-generated fragments were separated on precast 2% agarose gels containing ethidium bromide (E-Gel, Invitrogen) and band intensity determined (Kodak, Rochester, NY, USA). Relative band intensities were calculated by dividing total gene of interest band intensity by 18S rRNA band intensity (internal standard), and fold change in expression was determined by dividing SP-treated relative band intensity by mean saline (control) relative band intensity. Data represent the mean ± SEM of two separate PCR reactions per experimental animal. Reaction without reverse transcriptase served as a negative control.

### 2.7. Confocal Microscopy

Frozen bladder sections (14 *μ*m; intact bladders) were exposed to CD74 antiserum (sc-5438, specific for extracellular domain, Santa Cruz Biotechnology) and visualized using secondary antiserum labeled with FITC or TRITC. Control experiments included omission of the primary antiserum.

Confocal microscopy for cell-surface CD74 was performed with a Zeiss LSM 510 laser scanning microscope (Thornwood, NY, USA) with Argon laser (for excitation at 488 nm) and HeNe1 laser (for excitation at 543 nm). The slide was mounted on a Zeiss-inverted microscope (Axiovert 100 M) with either Plan-Neofluar 40×/0.75 N.A. air objective or Plan-Apochromat 100×/1.4 N.A. oil objective. For each group of experiments, identical settings for the imaging collection were used (via Reuse function). All the confocal images were collected and analyzed using Zeiss software either provided with the confocal imaging system or downloaded from Zeiss website (AxioVision LE, http://www.zeiss.com/C12567BE0045ACF1/Contents-Frame/3F3821B370EFC91CC125734C002FB38C).

## 3. Results

### 3.1. In Vivo Biotinylation of Urothelial Cell Surface Proteins

**Figure 1:**
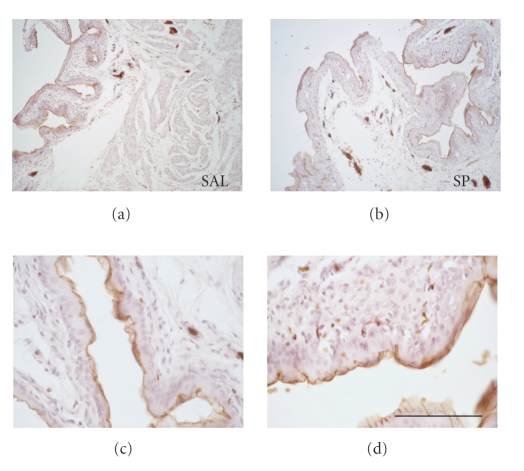
In vivo biotinylation of urothelium. (a) and (c) saline-treated bladders, (b) and (d) SP-treated bladders. Biotin labeling is determined only with strepavidin-HRP and is localized to surface urothelium. Calibration bar—(a), (b) = 400 *μ*m; (c), (d) = 100 *μ*m.

**Figure 2:**
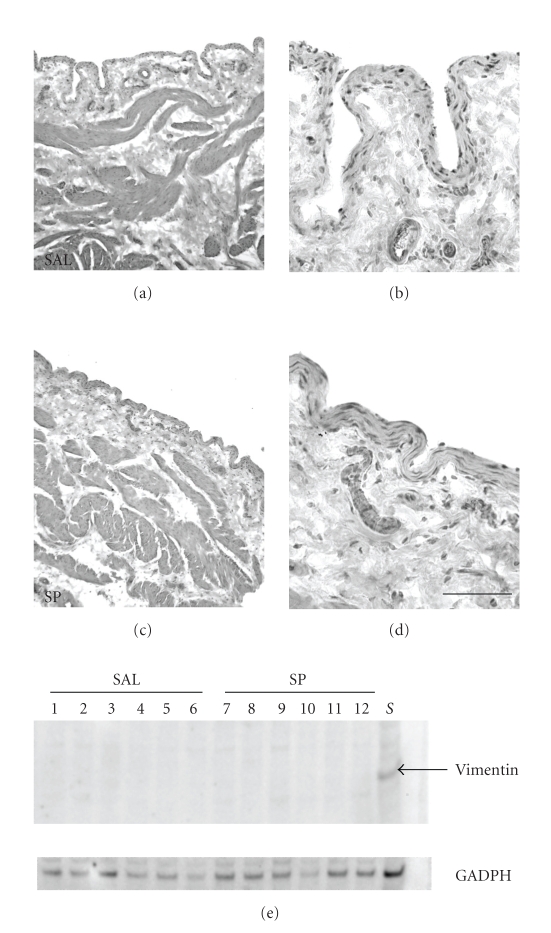
Epithelial aggregate separation and isolation (EASI) of rat bladder urothelium. Hematoxylin staining of scraped bladders. (a) and (b) saline-treated bladders, (c) and (d) SP-treated bladders. EASI only removes urothelium leaving intact lamina propria. Calibration bar—(a), (c) = 400 *μ*m; (b), (d) = 100 *μ*m. (e) Vimentin Western blot of total urothelial cell lysates. Total protein from isolated urothelial cells was separated on 4–12% bis tris acrylamide gels and transferred to PVDF. Lanes 1–6 saline-treated animals; lanes 7–12 SP-treated animals, lane S—10 *μ*g of scrapped bladder protein (positive control). Vimentin Western blot was stripped and reprobed with GAPDH which served as a loading control.

We developed a methodology to identify cell-surface protein expression in the urothelium using in vivo biotinylation and also determined urothelial cell-surface protein changes induced by SP treatment. In both treatment and control bladders, the biotinylation reaction occurred exclusively at the umbrella cell layer (Figures 1(a)–1(d)) indicating that only luminal cell-surface proteins were labeled with biotin. Bladder pieces remaining following EASI were stained with hematoxylin (Figures 2(a)–2(d)). As seen in both treatment and control bladders, our procedures resulted in the collection of only urothelial cells with the lamina propria and smooth muscle tissue in the bladder remaining intact (Figures 2(a)–2(d)). In addition, Western blotting of urothelial lysates for vimentin (fibroblast marker) from all twelve animals was negative, while present in the remaining (scraped) bladder pieces (Figures 2(e)), indicating that lamina propia fibroblasts were not included in the isolated cells. Therefore, these results suggest that our procedures successfully isolated urothelial cells without contamination (as determined by histology and vimentin Western blotting) from other cells in the lamina propia.

### 3.2. SP-Induced Changes in Urothelial Cell-Surface CD74 and MIF

**Figure 3:**
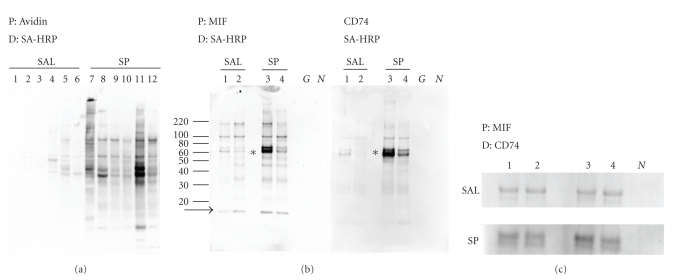
Expression of CD74 and MIF on urothelial bladder surface. (a) Total biotinylated protein. Isolated urothelial cells were lysed, biotinylated proteins were isolated by avidin agarose affinity chromatography, separated by 4–12% bis tris acrylamide gels electrophoresis, transferred to PVDF and biotin containing protein bands visualized with strepavidin-HRP only, no antibodies were used. P: indicates precipitation of proteins with avidin agarose, D: indicates detection with SA-HRP only, no antibodies were used. Lanes 1–6, saline-treated animals; lanes 7–12 SP-treated animals. (b) Immunoprecipitation of biotinylated CD74 or MIF. Isolated urothelial cells were lysed, 1 mg of total biotinylated proteins was isolated by avidin agarose affinity chromatography and biotinylated CD74 or MIF protein was immunoprecipitated using appropriate antibodies. Precipitates were separated by denaturing-reducing SDS PAGE and CD74 or MIF protein detected by strepavidin-HRP. P: indicates precipitation of proteins with MIF antibody (left panel) or CD74 antibody (right panel). D: indicates detection with SA-HRP only (left and right panel), no antibodies were used. Lanes 1, 2 saline-treated animals (number 4 and 5 from Figure 3(a)), lanes 3, 4 SP-treated animals (numbers 9 and 10 from Figure 3(a)). G: indicates immunoprecipitation with GAPDH antibody documents that only biotinylated proteins were immunoprecipitated, N: indicates immunoprecipitation with nonspecific goat IgG documents antibody specificity. Lines and numbers to the far left indicate the position of molecular weight markers. Asterisk indicates the location of 76 kDa band. Arrow indicates the location of 12 kDa uncomplexed MIF. (c) Coimmunoprecipitation of cell-surface MIF/CD74 complexes. Isolated urothelial cells were lysed, 1 mg total protein was used to purify biotinylated proteins by avidin agarose affinity chromatography. Biotinylated MIF proteins were precipitated with an anti-MIF antibody. Precipitates were separated by denaturing-reducing SDS PAGE and CD74 protein containing bands identified using anti-CD74 antibody followed by an antigoat-HRP. P: indicates precipitation of proteins by MIF antibody. D: indicates detection with CD74 primary antibody and antigoat HRP secondary antibody. Upper panel saline-treated animals, Lanes 1, 2, 3, 4, (numbers 1, 2, 3, and 6 from Figure 3(a)). Lower panel SP-treated animals, Lanes 1, 2, 3, 4, (numbers 7, 8, 11, and 12 from Figure 3(a)). N: indicates immunoprecipitation with nonspecific goat IgG documents antibody specificity.

Biotinylated proteins from isolated urothelial cells were purified by avidin agarose and 15 *μ*L of the fivefold concentrated eluate separated by reducing SDS-PAGE. The total amount of cell-surface biotinylated protein from each was quantified by determining total band intensity in each lane. Cell-surface protein as measured by biotinylated band intensity increased tenfold (*P* = .002, Mann-Whitney) following SP treatment (lanes 7–12, Figure 3(a); total intensity 68340 ± 23710 arbitrary units) compared with control animals (lanes 1–6, Figure 3(a); total intensity 6600 ± 2725 arbitrary units).

CD74 and MIF bands could not be detected by Western blotting of equal aliquots of avidin-purified urothelial cell lysates (data not shown), suggesting that the protein concentration was below the detection limit of this assay. Therefore, to test the relationship between relative amounts of cell surface MIF/CD74 and SP treatment, avidin-purified biotinylated proteins from urothelial lysates were immunoprecipitated with MIF or CD74 antibodies and detected using HRP-labeled strepavidin. SP treatment increased the amount of cell-surface CD74 as evidenced by increased amount of immunoprecipitable biotinylated CD74 from SP-treated urothelial cells (*P* = .03, Mann-Whitney; right panel, lanes 1, 2, animals 4, 5; lanes 3, 4, animals 9, 10; Figure 3(b)). In addition, the amount of cell surface (biotinylated) immunoprecipitable MIF was increased with SP treatment (*P* = .03, Mann-Whitney; left panel, lanes 1, 2, animals 4, 5; lanes 3, 4, animals 9, 10; Figure 3(b)). Not all the MIF was complexed to the 76 kDa band since 12 kDa MIF was found in the MIF immunoprecipitation (Figure 3(b), left panel, arrow). Immunoprecipitation with GAPDH was used to confirm that only biotinylated proteins were present as indicated by the absence of SA-HRP reactive bands (Figure 3(b), lane G, left and right panels). Goat IgG was used as a control of antibody specificity (Figure 3(b), lane N).

Both CD74 and MIF immunoprecipitation reactions showed a biotinylated (cell-surface) protein band at the same molecular weight (76 kDa), which could be attributed to a cell-surface CD74-MIF complex that is not broken upon reducing conditions (asterisk Figure 3(b)). Therefore, co-immunoprecipitation of cell-surface proteins with MIF antibody followed by CD74 Western blotting was used to determine if CD74 was associated with immunoprecipitable biotinylated (cell-surface) MIF. In addition co-immunoprecipitation was used to determine if SP treatment increased the amount of this complex. SP treatment increased the amount of this complex greater than twofold (*P* = .02, Figure 3(c) saline lanes 1–4, animals 1, 2, 3, 6, total intensity 32733 ± 17311 arbitrary units; SP lanes 1–4, animals 7, 8, 11, 12, total intensity 13956 ± 2505 arbitrary units). These results suggest that SP treatment increased availability of cell-surface CD74 receptor for MIF binding.

**Figure 4:**
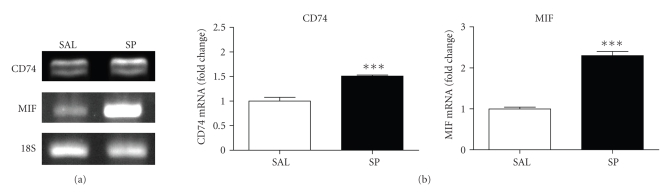
(a) MIF and CD74 mRNA expressions in isolated urothelial cells. Total RNA was isolated from urothelial cells and CD74 or MIF-specific primers used to amplify mRNA by endpoint PCR. A CD74 and MIF mRNA is increased in isolated urothelial cells after SP treatment. Data are representative of 5 salines and 5 SP-treated animals. 18S rRNA was used as a control for the amount of total RNA added to each reaction tube. (b) Relative expression of CD74 and MIF in isolated urothelial cells. Data are expressed as fold change calculated by determining mean gene expression ratio (net intensity of gene-specific band divided by net intensity of 18S rRNA band) normalized to saline controls. (***P < .001, *n* = 5).

Along with increased cell-surface CD74 and MIF SP-treatment also resulted in increased gene expression of both CD74 and MIF within isolated urothelial cells (Figure 4).

**Figure 5:**
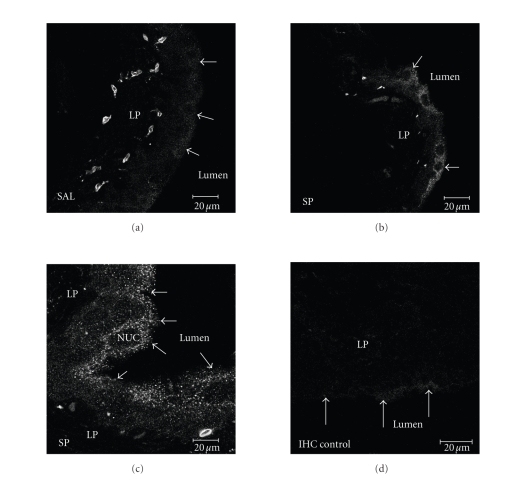
CD74 confocal microscopy. Frozen bladder sections (14 *μ*m; intact bladders) were exposed to CD74 antiserum and visualized using secondary antiserum labeled with FITC or TRITC. Control experiments included omission of the primary antiserum (panel D). (a) Saline-treated bladder with 40× objective. (b) SP-treated bladder with 40× objective. (c) SP-treated bladder with 100× objective. (d) Saline-treated bladder with 40× objective, secondary antibody only. Excitation wavelength of 488 nm wasused and emission wavelength of 505 nm and up was collected. Frame size of the image for (a), (b), and (d) is 230.3 *μ*m × 230.3 *μ*m; optical thickness of the images is 2 *μ*m. Frame size of the image for (c) is 92.1 *μ*m × 92.1 *μ*m; optical thickness of the image is 0.9 *μ*m. LP: lamina propria; NUC: nucleus; arrows indicate luminal surface of urothelial cells.

Finally, in order to confirm that CD74 localizes to the bladder urothelial cell surface and that cell-surface expression is increased with SP treatment, nonpermeabilized bladder tissue was immunostained using CD74 antibody (Santa Cruz, sc-5438) and examined by confocal microscopy. CD74 antibody detected expression in a subpopulation of these cells. As seen in Figure 5, CD74 protein expression is localized to the cell surface and is increased with SP treatment (Figures 5(b), 5(c)). Control experiments included tissue not exposed to secondary antibody (Figure 5(d)).

## 4. Discussion

MIF is a constitutively expressed, multifunctional cytokine that functions to counterregulate the effects of glucocorticoids and to stimulate the synthesis and secretion of other proinflammatory mediators such as TNF-*α* and IL-1*β*, as such it has been suggested that MIF is a key initiator of the inflammatory cascade [21, 22]. In addition to its function in the inflammatory response, MIF functions as an autocrine regulator of cell proliferation and differentiation. Serum-starved NIH/3T3 cells secreted endogenous MIF as a consequence of serum stimulation resulting in proliferation of quiescent cells. This response was associated with phosphorylation and subsequent activation of ERK 1/2 [23].

SP is an afferent nerve fiber neurotransmitter that mediates inflammation by inducing vasodilatation and plasma extravasation [24]. SP has a short half-life suggesting that inflammation initially induced by SP is maintained by the release of other proinflammatory mediators [24]. Our previous experiments established MIF as one such SP-induced proinflammatory mediator [4]. SP affects the rat bladder by upregulating MIF expression and inducing the release of urothelial MIF into the bladder lumen [4]. Sequestering the MIF released into the bladder lumen with anti-MIF antibodies decreases SP-induced inflammatory changes in the bladder [8]. In addition, our studies have shown that the MIF receptor, CD74, is also found in the urothelium and that SP increases CD74 protein and mRNA amounts in the bladder [15]. The findings of this study show that this increase in MIF and CD74 mRNA is localized to the urothelium. Thus, we propose that MIF, constitutively expressed by the urothelium and upregulated in SP-induced inflammation, binds to cell-surface CD74 receptor, which perpetuates inflammation. Direct evidence of SP-induced MIF-CD74 interaction had not been described previously. The aim of the present experiments was to determine SP-induced cell-surface CD74 expression and the interaction of cell-surface CD74 with MIF in the rat urothelium.

It is difficult to account for the absence of an MIF response under “normal” physiological conditions considering the ubiquitous expression of this protein [25]. MIF is readily detected in urine suggesting that bioactive MIF is readily available in the local environment [2, 4]. CD74 amounts within the normal bladder are present in low amounts and our previous immunoprecipitation studies demonstrated increased MIF-CD74 complexes as a result of SP-induced inflammation, suggesting that it is likely CD74 availability controls the cellular response to exogenous MIF [15]. Interestingly, we found that CD74 expression in SP-treated bladder is limited largely to the urothelial cell layer surface, a differentiated cell type believed to play a role in maintaining the integrity of the urothelium (Figure 5).

Under normal physiological conditions, urothelial cells that do not express cell-surface CD74 (or express small amounts of CD74) would not be able to respond to secreted MIF via the well-described CD74-mediated ERK-activation. The results presented here suggest that increased cell-surface expression of CD74 induced by SP results in greater binding of secreted MIF molecules by CD74, and therefore greater activation of CD74-mediated signaling pathways (e.g., ERK-activation). In particular, the results of the immunoprecipitation experiments described here establish that SP treatment results in an increased cell-surface CD74/MIF complex when compared with saline controls (Figure 3(c)). Stable immunoprecipitable CD74 complexes of similar molecular weights to what we report here have been identified previously in both human peripheral blood T-cells and duodenal epithelial cells [26, 27]. The exact nature and function of these complexes was not established.

In their entirety, these results suggest that the relative contributions of MIF to normal bladder function are minimal, but inflammatory processes are reliant upon bioactive MIF and cell-surface expression of the MIF receptor, CD74. This would imply that pharmacologic inhibition of MIF (e.g., using pharmacological antagonists of MIF tautomerase activity with ISO-1) would have little effect on normal bladder function, but may prove to be a potent bladder inflammatory inhibitor. As evidenced by the data presented here, a model for MIF response at the cellular level emerges: under normal conditions although MIF is present in the surrounding tissues as well as in the peripheral blood; the cell-surface receptor is absent. In response to inflammatory stimuli, epithelial cells express the MIF receptor (CD74) on their surface, thus initiating inflammatory response.

## 5. Conclusions

In summary, the present study documents increased MIF and urothelial cell-surface CD74 expression during inflammation. The emerging role of MIF in bladder inflammation suggests that modulating this cytokine activity may result in new, selective, and therapeutic modalities.
